# Digital Scan‐Guided Endodontic Management of Obliterated Canals in Maxillary Central Incisors: A Precision‐Based Minimally Invasive Clinical Report of Two Cases

**DOI:** 10.1155/crid/5762976

**Published:** 2026-06-08

**Authors:** Kishan Paul Raja, Shravan Kini, S. Vidhyadhara Shetty, Samreena Kalander, K. Rahul Pai

**Affiliations:** ^1^ Department of Conservative Dentistry and Endodontics, Yenepoya Dental College, Yenepoya (Deemed to be University), Mangalore, Karnataka, India, yenepoya.edu.in

**Keywords:** 3D printed guide, CBCT, digital workflow, guided endodontics, pulp canal obliteration

## Abstract

Pulp canal obliteration (PCO) presents a significant clinical challenge in endodontics due to progressive canal calcification, loss of identifiable anatomy, and increased risk of iatrogenic errors during access preparation. Conventional techniques for canal localization may lead to excessive dentin removal and structural compromise. Guided endodontics has emerged as a minimally invasive approach that combines cone beam computed tomography (CBCT) with digital intraoral surface scanning for virtual planning and fabrication of a three‐dimensional printed access guide. This case report describes the guided endodontic management of two maxillary central incisors with extensive canal calcification and associated periapical pathology. Limited‐field CBCT, intraoral scanning, and software‐based guide design enabled conservative and precise access preparation. The guide ensured accurate angulation, controlled depth, and alignment with the long axis of the root while preserving tooth structure. Both cases demonstrated favorable clinical and radiographic outcomes on follow‐up, supporting the predictability and clinical relevance of guided endodontics.

## 1. Introduction

Pulp canal obliteration (PCO) or calcific metamorphosis occurs due to progressive deposition of hard tissue within the pulp chamber, resulting in loss of the canal lumen. This phenomenon is commonly noted in traumatic dental injuries and also occurs secondary to certain orthodontic treatment, aging, restorative procedures, and pulpal irritation [1–3]. On histological investigation, PCO involves the deposition of tertiary dentin and mineralization in the canal space, leading to obliteration of the canal [4]. Clinically, PCO is associated with tooth discoloration and decreased response to pulp vitality testing. Even though the teeth remain asymptomatic, it may lead to periapical pathology, which requires endodontic intervention. The management of these cases poses challenges because of their lack of identifiable canal orifices, change in the internal anatomy, and decreased tactile feedback during endodontic instrumentation [5].

Traditional periapical radiography provides only two‐dimensional information and fails to describe the buccolingual extent of calcification due to anatomical superimposition [6]. In calcified anterior teeth, especially maxillary central incisors with thin cortical plates, access preparation carries the risk of labial perforation or more removal of dentin [6].

Cone beam computed tomography (CBCT) has potentially developed three‐dimensional visualization of root canal morphology and periapical structures. The true advancement in managing calcified canals lies in the integration of CBCT with digital surface scanning for guided endodontic access planning [7]. It involves merging volumetric CBCT data with intraoral surface scans to create a virtual 3D model. Using software, a trajectory is designed to follow the canal path while preserving dentin. A customized 3D printed guide is then fabricated to run the bur precisely in the predetermined path [8].

This digital approach decreases the operator dependency, improves accuracy, and reduces structural compromise. Compared to traditional endodontic techniques, guided endodontics has demonstrated greater precision in localization of the canal, reduced dentin removal, and decreased incidence of perforation. [9, 10].

The present case report explains the comprehensive digital workflow and guided endodontic management of two maxillary central incisors with extensive canal obliteration. Emphasis is on virtual planning, fabrication of stent, and clinical execution of guided access preparation.

## 2. Case Reports

### 2.1. Case Report 1

A 42‐year‐old male patient with the chief complaint of discolored maxillary right central incisor for the past 20 years. History reveals the presence of dental trauma before 20 years without pain, swelling and sinus discharge. His medical history was noncontributory.

### 2.2. Clinical Examination

Intraoral examination revealed marked yellowish discoloration of Tooth 11. The tooth was asymptomatic and exhibited no mobility. Periodontal probing depths were within normal limits. Thermal and electric pulp testing elicited no response, suggesting loss of pulpal vitality.

### 2.3. Radiographic and CBCT Evaluation

A limited field‐of‐view CBCT scan (40 × 50 mm) of the maxillary anterior region was obtained to assess internal anatomy and periapical status.

CBCT analysis revealed poor visualization of the canal lumen extending from the coronal region up to approximately 14.04 mm from the incisal edge. The total tooth length measured 20.42 mm with loss of lamina dura associated with an ill‐defined radiolucency measuring 2.10 mm mesiodistally and 1.20 mm superoinferiorly. Discontinuity of the labial cortical plate at the level of the lesion was noted.

These findings confirmed PCO with associated periapical rarefying osteitis. The clinical presentation, radiographic findings, CBCT evaluation, and virtual drill path planning are shown in Figure 1.

**Figure 1:**
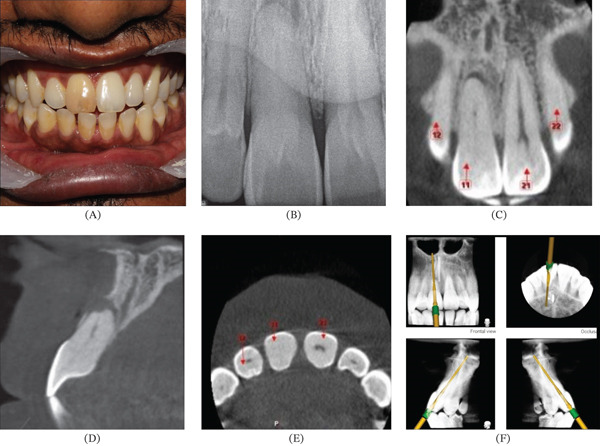
(A) Preoperative intraoral photograph showing discoloration of the right maxillary central incisor. (B) Preoperative intraoral periapical radiograph demonstrating pulp canal obliteration with associated periapical radiolucency. (C) CBCT image (coronal view) with respect to Tooth #11. (D) CBCT image (sagittal view) with respect to Tooth #11. (E) CBCT image (axial view) with respect to tooth #11. (F) Virtual planning showing CBCT–intraoral scan alignment and guided access path design along the tooth′s long axis.

After discussing the treatment plan, potential risks, and benefits of guided endodontic therapy, written informed consent was obtained.

### 2.4. Digital Workflow and Guide Fabrication

An intraoral optical scan of the maxillary arch was obtained using a TRIOS (3Shape) scanner. The surface scan was exported as an STL file and merged with the DICOM data from CBCT using AIS software. A digital drill of size 1 mm diameter and 22 mm length was superimposed on the canal trajectory which aligns with the long axis of the root. Then the pathway was evaluated in axial, sagittal, and coronal sections to attain optimal angulation, depth control, and elimination of cortical perforation. The guide design was exported as an STL file and fabricated using a NextDent 3D printer with NextDent resin, incorporating a metal guiding sleeve to ensure controlled bur angulation and depth, while extending onto adjacent teeth for stability.

### 2.5. Clinical Procedure

Initially, the fit and passive stability of the guide were intraorally verified. Anesthesia was given. After rubber dam isolation, the guide was then positioned and stabilized manually. Access preparation was initiated under microscopic magnification using a long‐shank tapered round‐end diamond bur (ISO 199‐016; Mani), with a maximum head diameter of 1.6 mm and an approximate tip diameter of 0.8–1.0 mm. Microscopic magnification enhanced visualization of the guided entry point, enabled continuous assessment of dentin removal, and aided in identifying calcified canal remnants while minimizing the risk of deviation or perforation during guided access preparation. The bur, with a working length of 10.2 mm, was used to perform the initial punch cut through enamel and extend into superficial dentin up to the preplanned entry point. This was followed by the introduction of an EG 5 bur (SS White) along the guided trajectory to advance the preparation deeper through dentin toward the calcified canal space. The EG 5 bur was used in accordance with the predetermined angulation and depth derived from digital planning. The EG 5 bur was specifically selected for its tapered microendodontic design, which allows precise and conservative dentin removal while preserving peri‐cervical tooth structure and facilitating guided negotiation of calcified canals.

Upon reaching the predetermined depth, canal negotiation was initiated using #6, #8, and #10 K‐files, followed by D‐Finder files to establish patency. After successful negotiation, a glide path was created. Rotary instrumentation was performed up to size 25 with 4% taper under copious irrigation with 5.25% sodium hypochlorite. Due to procedural duration, intracanal medicament was placed, and obturation was deferred. At the subsequent visit, the irrigation protocol included three cycles of ultrasonically activated 5.25% NaOCl (20 s each), followed by 17% EDTA for 1 min and final irrigation with 2% chlorhexidine. Obturation was completed using size 25/4% gutta‐percha cones with AH Plus sealer. The tooth was restored and scheduled for follow‐up. At the 6‐ and 12‐month evaluations, the patient remained asymptomatic, and radiographs demonstrated significant reduction of the periapical lesion and zirconia crown was delivered at subsequent visits (Figure 2).

**Figure 2:**
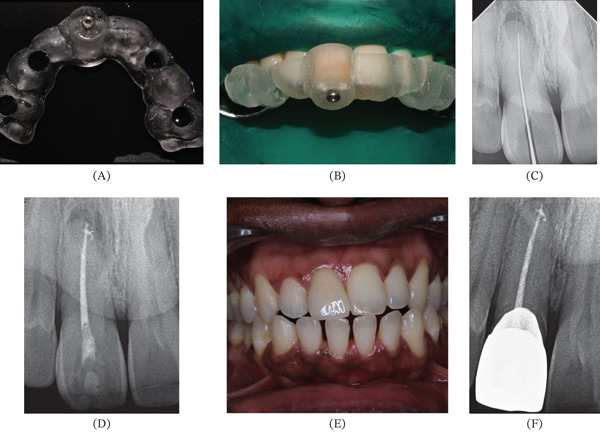
(A) Three‐dimensional printed surgical guide fabricated based on digital planning. (B) Intraoral placement of the guided template ensuring proper fit and stabilization. (C) Working length determination radiograph following guided access and canal negotiation. (D) Immediate postoperative radiograph after obturation. (E) Postoperative intraoral photograph at follow‐up after placement of a zirconia crown. (F) Twelve‐month follow‐up radiograph demonstrating significant periapical healing.

### 2.6. Case Report 2

A 25‐year‐old female patient reported with the chief complaint of discoloration of the maxillary right central incisor (11) for approximately 5 years, with no associated pain or swelling. Medical and dental histories were noncontributory. Clinical examination revealed discoloration of the affected tooth, showing nonresponsivity to the pulp sensitivity test. Surrounding soft tissues appeared normal.

### 2.7. CBCT Findings

CBCT imaging revealed extensive PCO, characterized by complete obliteration in the cervical third and marked obliteration of the middle third, with partial canal patency preserved in the apical third. The total tooth length measured 20 mm. Hyperdense calcific deposits were evident in both the cervical and apical thirds of the canal space. Additionally, flattening of the root apex was observed, a feature consistent with external root resorption.

The final diagnosis established was PCO associated with external root resorption. The clinical presentation, radiographic findings, CBCT evaluation, and virtual guided planning are shown in Figure 3.

**Figure 3:**
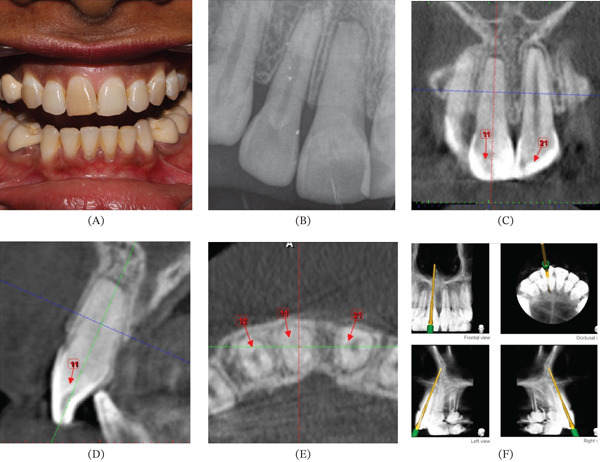
(A) Preoperative intraoral photograph showing discoloration of the right maxillary central incisor. (B) Preoperative intraoral periapical radiograph demonstrating pulp canal obliteration with respect to Tooth #11. (C) CBCT image (coronal view) with respect to Tooth #11. (D) CBCT image (sagittal section) with respect to Tooth #11. (E) CBCT image (axial section) with respect to Tooth #11. (F) Virtual planning images showing digital alignment of CBCT data with intraoral scan and design of the guided access trajectory.

### 2.8. Guided Endodontic Procedure

Intraoral scanning and CBCT data merging were done. A virtual drill path was planned centrally within the calcified canal space. Multiplanar assessment confirmed correct angulation and depth. A 3D printed guide was fabricated and verified intraorally. Access preparation was initiated using the same long‐shank tapered round‐end diamond bur (ISO 199‐016; Mani; maximum diameter 1.6 mm, tip diameter approximately 0.8–1.0 mm, working length 10.2 mm) under microscopic magnification to achieve initial enamel penetration and superficial dentin removal up to the planned entry point. Subsequently, a custom lab‐manufactured drill (1.2 mm diameter) was introduced along the guided path to extend the preparation deeper into dentin following the predetermined trajectory and depth. This facilitated precise access toward the calcified canal space, after which canal negotiation was performed using small K‐files to establish patency.

Upon reaching the planned depth, canal negotiation was performed using #6, #8, and #10 K‐files, followed by D‐Finder files to establish patency. Glide path and rotary instrumentation up to size 25/4% were completed under sodium hypochlorite irrigation. The irrigation protocol and obturation technique are similar to those in Case 1.

Postoperative imaging showed adequate canal obturation. The patient remained asymptomatic at follow‐up and nonvital bleaching was done for the tooth on subsequent visits. The guided access workflow, intraoperative procedures, and postoperative obturation outcome are presented in Figure 4.

**Figure 4:**
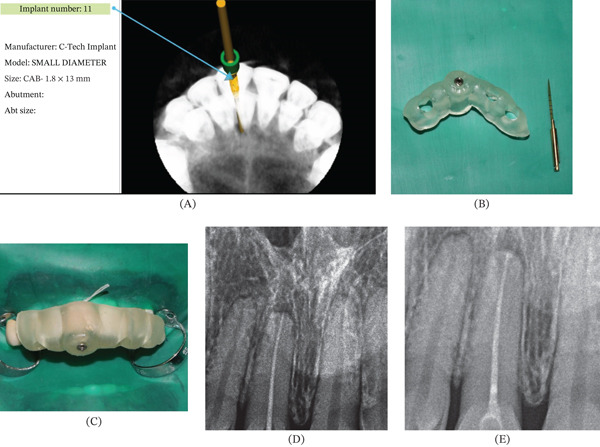
(A) Virtual implant planning software interface demonstrating the planned drill path aligned along the long axis of Tooth #11. (B) Three‐dimensional printed endodontic guide with corresponding guided drill. (C) Intraoral placement of the guide under rubber dam isolation confirming proper seating and stability. (D) Working length determination radiograph following guided access and canal negotiation. (E) Immediate postoperative radiograph after obturation.

## 3. Discussion

PCO is a common sequela of trauma, aging and chronic pulpal irritation, which ultimately results in the progressive calcification within the canal space [1–3]. Calcified teeth often result in pulpal necrosis and certain periapical pathology, which requires endodontic management. The clinical challenge is due to difficulty in canal location and negotiation without compromising the structural integrity. The complications due to increased removal of dentin, deviation from the original trajectory, and perforation of root are potentially increased in calcified anterior teeth [4].

Accurate assessment of root canal morphology, residual canal patency, and periapical status is fundamental to determining prognosis and treatment strategy. Traditional two‐dimensional radiographs give insufficient information regarding the calcification of the root canal because of superimposition and depiction of buccolingual dimension [5, 6]. Patel et al. [7] and Cotton et al. [11] highlighted the limitations of it and emphasized the requirement of three dimensional imaging in complex cases. However, although CBCT undoubtedly enhances visualization of internal anatomy, its role in contemporary practice extends beyond diagnosis alone.

In our cases, CBCT imaging provided a complete assessment of canal calcification, locating the canal patency, and evaluating the cortical structures. In Case 1, CBCT revealed the cortical plate involvement and the canalis sinuosus proximity, apart from the degree of obliteration. Similarly, in Case 2, CBCT showed differentiation between partial and complete obliteration and facilitated the identification of external root resorption which is in accordance with findings of Patel et al. [12] and Durack and Patel [13]. Accurate identification of anatomical and pathological features is important for safe access strategy.

The advancement of PCO depends on the transformation of CBCT data from diagnosis to treatment planning. Guided endodontics provides more accurate access preparation, which does not depend on operator experience and magnification, with the integration of CBCT and intraoral scanning permitting accurate planning of the access pathway [13].

Krastl et al. [14] explained the importance of predictable approach for treating calcified canals showing apical pathology with minimal iatrogenic complications. Connert et al. [15] in their study introduced the concept of microguided endodontics and explained decreased dentin removal when compared to traditional access techniques. Buchgreitz et al. [8] confirmed the clinical significance and reproducibility of guided canal preparation in the affected teeth.

The digital workflow in the present cases starts with acquisition of limited field of view CBCT data along with intraoral scanning. Combining DICOM and STL data enabled fabrication of a comprehensive three‐dimensional model exhibiting both internal and external anatomy. This merging is significant in calcified anterior teeth. Virtual drill planning allowed perfect alignment along the long axis of the root with preexisting angulation and depth control. Multiplanar assessment leads to the elimination of cortical perforation and anatomical structures. Followed by the fabrication of a customized 3D printed guide, this digital plan was transformed into a clinical procedure. By mechanically directing the bur along a trajectory, the guide reduced operator variability during canal localization.

The anatomical considerations in maxillary central incisors justify the requirement for guided access. These teeth often have thin labial dentin walls and labial cortical bone. In severe calcification, access preparation carries a risk of labial perforation, when the operator tries to locate the canal blindly. The guided template eliminates this risk by maintaining a controlled path toward the canal location. A systematic review by Peña‐Bengoa et al. [16] described the effectiveness of guided endodontics in enhancing canal localization accuracy and minimizing complication during the procedure. The review described that guided approaches potentially enhance predictability compared to conventional techniques, especially in anterior teeth with PCO.

In both cases, the guided stent offered conservative access preparation. Canal negotiation was achieved without structural compromise, without any procedural errors. This technique improved clinician confidence, decreased exploratory time, and preserved dentin. It is essential to recognize that guided endodontics should not be seen as an adjunct but rather as a definitive strategy in managing calcified canals. Although CBCT provides diagnostic insight, the true value is recognized when incorporated into a comprehensive digital workflow resulting in guide fabrication. This ensures that three‐dimensional imaging directly informs clinical practice.

Guided endodontics aligns with the principles of minimally invasive endodontics. Restricting dentin removal leads to unnecessary exploration and preservation of structural integrity. This is important in anterior teeth, where aesthetic and functional considerations is mandatory [17, 18]. The present case report reinforces the concept that successful management of PCO relies on both advanced imaging and application of digital planning protocols. The synergy between CBCT, intraoral scanning, virtual planning, and 3D printing represents an advancement in contemporary endodontic practice.

These findings support previously published evidence promoting guided endodontics as a predictable, safe, and minimally invasive approach for managing calcified maxillary central incisors [17, 18]. Adoption of this digital workflow can potentially reduce iatrogenic risks, enhance treatment precision, and improve long‐term clinical outcomes in complex endodontic cases.

## 4. Conclusion

Guided endodontic therapy, along with CBCT‐based planning and digital intraoral scanning, gives a precise, minimally invasive, and predictable solution for managing calcified maxillary central incisors. The digitally designed stent ensures accurate canal localization, access preparation, and preservation of tooth structure. Incorporation of guided endodontics into clinical practice reduces iatrogenic risks and improves treatment outcomes in complex cases of PCO [19].

## Funding

No funding was received for this manuscript.

## Consent

Written informed consent was obtained from the patient for publication of this case report and accompanying clinical images. The patient was informed that all efforts would be made to maintain anonymity; however, complete anonymity cannot be guaranteed.

## Conflicts of Interest

The authors declare no conflicts of interest.

## Data Availability

The data that support the findings of this study are available on request from the corresponding author. The data are not publicly available due to privacy or ethical restrictions.
